# Supplementary material for: Multimodal deep learning using on-chip diffractive optics with in situ training capability
Source: Nat Commun. 2024 Jul 23;15:6189. doi: 10.1038/s41467-024-50677-3 (PMC11266606; doi:10.1038/s41467-024-50677-3)
Supplement: Supplementary file 1 — Supplementary Information [file 41467_2024_50677_MOESM1_ESM.pdf]

Supplementary Information for

Multimodal deep learning using on-chip diffractive optics with *in situ* training capability

Junwei Cheng[1], Chaoran Huang[2], Jialong Zhang[1], Bo Wu[1], Wenkai Zhang[1], Xinyu Liu[1], Jiahui Zhang[1], Yiyi Tang[1], Hailong Zhou[1], Qiming Zhang[3], Min Gu[3], Jianji Dong[1,4]*, and Xinliang Zhang[1,4]

[1]*Wuhan National Laboratory for Optoelectronics, Huazhong University of Science and Technology, Wuhan 430074, China*

[2]*Department of Electronic Engineering, The Chinese University of Hong Kong, Hong Kong 999077, China*

[3]*Institute of Photonic Chips, University of Shanghai for Science and Technology, Shanghai 200093, China*

[4]*Optics Valley Laboratory, Wuhan 430074, China*

**Correspondence: jjdong@mail.hust.edu.cn*

Contents

Supplementary Note 1. Photonic processor based on task inference....................2

Supplementary Note 2. The forward propagation model of TDONN....................3

Supplementary Note 3. The error backward propagation model of TDONN....................5

Supplementary Note 4. Design of the period of hidden layers....................6

Supplementary Note 5. Simulation of the TDONN....................7

Supplementary Note 6. Design details of the photonic-electronic prototype....................8

Supplementary Note 7. More tactile tasks for the TDONN chip....................10

Supplementary Note 8. Estimation of the latency of the TDONN chip....................10

Supplementary Note 9. Temperature stability of the TDONN....................12

Supplementary Note 10. Long-term stability of the TDONN....................13

Supplementary Note 11. Estimation of throughput, computing density, and energy consumption....................13

Supplementary References....................15

Supplementary Note 1. Photonic processor based on task inference

Photonic processor based on task inference is a new computing paradigm, and its comparison with the conventional matrix multiplication processor is shown in Supplementary Figure 1. The photonic processor based on matrix multiplication can only perform one matrix multiplication calculation per light propagation, requiring frequent O/E conversions. In contrast, the photonic processor based on task inference can accomplish one inference task per light propagation without the need for frequent O/E conversions, resulting in low latency and power consumption.

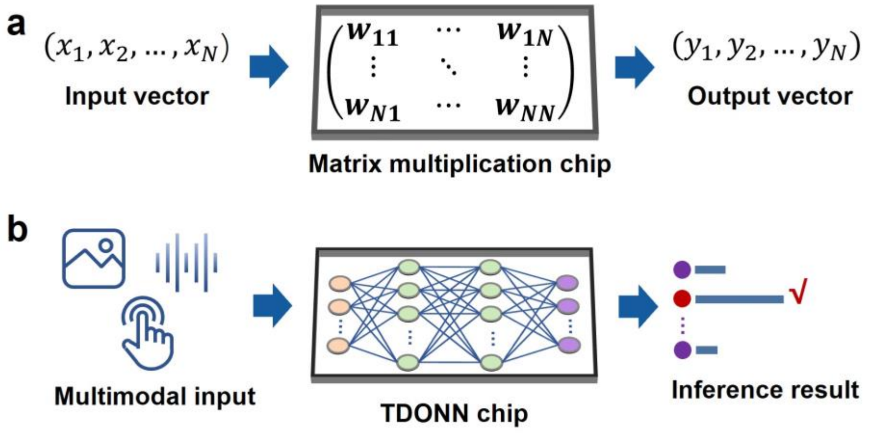

Supplementary Figure 1. Two different computing paradigms. a Photonic processor based on matrix multiplication. **b** Photonic processor based on task inference.

The workflows of these two types of photonic processors are shown in Supplementary Figure 2. Supplementary Figure 2a illustrates the workflow of traditional matrix multiplication processors, where the photonic chip is treated as a matrix kernel, and each light propagation can perform a matrix multiplication operation. This scheme represents precise computation, corresponding to the calculations in electrical neural networks. The computational process of deep neural network requires repeated invocation of the matrix multiplication processor, which is essentially a time-division multiplexing of matrix multiplication, resulting in significant delays. Additionally, frequent O/E conversions and digital-to-analog conversions will also lead to extra power consumption. Since a neural network model contains numerous matrix multiplications, it necessitates multiple loading operations of new input data and the utilization of the photonic processor to execute matrix multiplication operations. This computing paradigm poses a challenge: every matrix multiplication involves O/E conversion during the computation process. Another problem is data storage and recovery. In conventional architectures, the output data from the photonic processor is usually stored in electrical cache and need to be reshaped into the final inference result after all computations are completed.

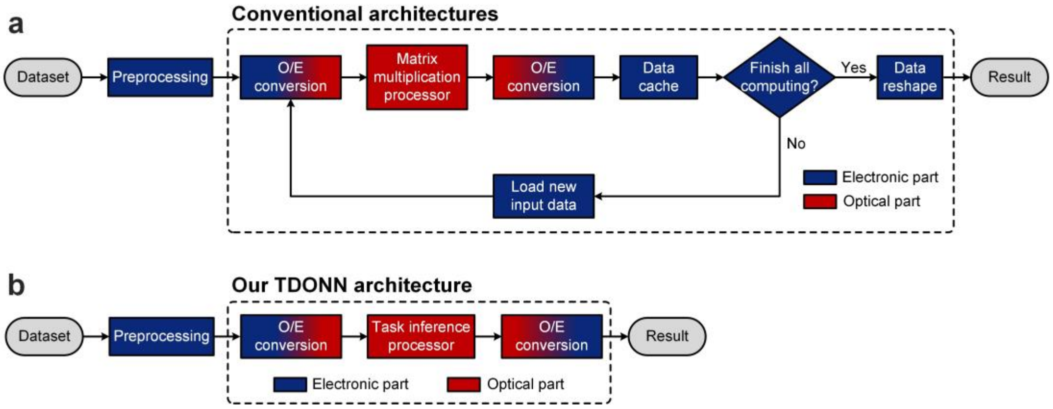

Supplementary Figure 2. The workflows of different photonic processors. a Photonic processor based on matrix multiplication. **b** Photonic processor based on task inference.

On the contrary, our TDONN architecture is a photonic processor architecture based on task-specific inference, and its workflow is shown in Supplementary Figure 2b. In this scheme, the photonic chip can be regarded as a "black box" functionally equivalent to an on-chip linear neural network, where each light propagation can perform an inference task. Unlike precise computation, this scheme is a form of fuzzy computation that can directly map the input space (feature vectors) to the output space (classification labels) by adjusting the trainable units on the photonic chip. By comparing the contents in the dashed boxes of the two types of workflows in Supplementary Figure 2, it can be seen that the TDONN architecture differs significantly from other photonic matrix multiplication schemes. Specifically, the TDONN architecture requires only one forward propagation of light and two O/E conversions during the inference process, and it eliminates the need for electrical data storage and reshape, which can effectively overcome the shortcomings of traditional matrix multiplication architectures.

Supplementary Note 2. The forward propagation model of TDONN

The forward propagation model (FPM) of the TDONN is designed based on the physical process of light propagation in a slab waveguide. In the input layer, the input data is modulated onto the intensity of the optical signal. The optical signal passes through the narrow waveguide into the slab waveguide, and then diffracts over a distance (i.e. the period of hidden layer) to reach the first hidden layer. Each hidden layer consists of multiple neurons to modulate the light field. In this simulation model, the amplitude transmission coefficient is 1, and the neurons only delay the phase. The optical signal passing through the first hidden layer then diffracts over another same distance to reach the next hidden layer, and this process repeats until the light exits from the last hidden layer. After diffracting for a distance, a certain light intensity distribution is finally formed in the output layer. Multiple detectors are arranged at equal intervals at the output layer. The number of detectors and the method to determine

the result can be designed according to the task requirements. For example, in classification task, the number of detectors is the total number of categories, and the category represented by the detector with the largest received light intensity is the predicted classification result.

The FPM is constructed using the Discrete Fourier Transform (DFT) method. The light field in the $(m+1)^{th}$ hidden layer is obtained by diffracting the output light from the mth hidden layer. Similar to the principle of angular spectrum method[1], the light field is decomposed in the spatial frequency domain, and different frequency components undergo different phase delays. All the frequency components are synthesized to obtain the target light field, which can be expressed by Eq. (S-1) and Eq. (S-2):

$$\mathbf{E}_{\mathrm{m+1}} = \mathbf{F}^{\dagger}\mathbf{P}_{\mathrm{m}}\mathbf{F}\mathbf{\Phi}_{\mathrm{m}}\mathbf{E}_{\mathrm{m}} \qquad (S-1)$$

$$\mathbf{P}_{\mathrm{m}}^{\xi,\xi} = exp\left(\mathrm{i}\frac{2\pi z_{\mathrm{m}} n_{\mathrm{m}}}{\lambda}\sqrt{1-\left(\lambda\nu_{\xi}/n_{\mathrm{m}}\right)^{2}}\right) \qquad (S-2)$$

where $\mathbf{E}_{\mathrm{m}}$ is the light field of the mth hidden layer, $\mathbf{F}$ is the DFT matrix, $\mathbf{F}^{\dagger}$ is the conjugate transpose of the DFT matrix. $\mathbf{\Phi}_{\mathrm{m}}$ is a diagonal matrix, whose diagonal elements are the transmission coefficients of the individual neurons in the mth hidden layer. Since the amplitude transmission coefficient is approximated to be 1, the transmission coefficient is the phase delay factor. $\mathbf{P}_{\mathrm{m}}$ is the diffraction matrix between the mth and the $(m+1)^{th}$ hidden layer, z_{m} is the diffraction distance, ξ is the row (column) number of the element, and ν_{ξ} is the corresponding spatial frequency.

Since the simulations in this work focus on light propagation in linear networks, the FPM model we use for simulation does not include nonlinear activation functions. If the nonlinear activation function module needs to be considered, the light field in the $(m+1)^{th}$ hidden layer can be rewritten as:

$$\mathbf{E}_{\mathrm{m+1}} = f(\mathbf{F}^{\dagger}\mathbf{P}_{\mathrm{m}}\mathbf{F}\mathbf{\Phi}_{\mathrm{m}}\mathbf{E}_{\mathrm{m}}) \qquad (S-3)$$

where $f(\ )$ is a nonlinear activation function, which can be flexibly designed according to different tasks. For linear systems, the nonlinear activation function can be set as a positive scaling function with a scaling factor of 1.

It should be mentioned that this theoretical model is intended to help non-expert readers understand the propagation of light in the diffractive neural network. However, the conditions of the actual experiments are not ideal, for example, the waveguide boundary will reflect some light, which will reach the next diffractive layer, thus the actual light propagation does not strictly follow this theoretical model.

Supplementary Note 3. The error backward propagation model of TDONN

The error backward propagation model (EBPM) is based on the gradient descent optimization algorithm. It calculates the gradients of the network parameters by the loss function and updates the parameters in the opposite direction of the gradients, thereby gradually reducing the loss during training and improving the performance of the optical neural network. When the target light field intensity distribution is $\mathbf{I}^{\mathrm{des}}$ and the actual light field intensity distribution is $\mathbf{I}$, the loss function L can be written as Eq. (S-4). The light intensity distribution $\mathbf{I}$ can be represented by $\mathbf{E}_{\mathrm{M+1}}$, as in Eq. (S-5). Since there are M hidden layers in total, the light field of the output layer is $\mathbf{E}_{\mathrm{M+1}}$.

$$\mathrm{L} = \left|\mathbf{I}-\mathbf{I}^{\mathrm{des}}\right|^2 \qquad (S-4)$$

$$\mathbf{I} = \mathbf{E}^{*}_{\mathrm{M+1}} \odot \mathbf{E}_{\mathrm{M+1}} \qquad (S-5)$$

where the symbol $\odot$ denotes the element-wise multiplication. The partial derivative of L with respect to the phase delay of the s_1^th^ neuron in the mth hidden layer can be calculated according to Eq. (S-6)

$$\frac{\partial L}{\partial \phi_{s_1}^{m}} = \sum_{s=1}^{S} \frac{\partial L}{\partial E_{s_1}^{M+1}} \frac{\partial E_{s_1}^{M+1}}{\partial \phi_{s_1}^{m}} = 4\Re\left\{\left[\boldsymbol{E}_{M+1} \odot \left(\mathbf{I}-\mathbf{I}^{\mathrm{des}}\right)\right]^{\dagger} \frac{\partial \boldsymbol{E}_{M+1}}{\partial \phi_{s_1}^{m}}\right\} \qquad (S-6)$$

where S is the number of neurons in each hidden layer, $E_{s_1}^{M+1}$ is the light field of the s_1^{th} neuron in the output layer, $\phi_{s_1}^{m}$ is the phase delay of the s_1^{th} neuron in the mth hidden layer, $\boldsymbol{E}_{M+1}$ is the output light field, $\mathbf{I}-\mathbf{I}^{\mathrm{des}}$ is the difference between output and target. Since $\boldsymbol{E}_{M+1}$ and $\mathbf{I}-\mathbf{I}^{\mathrm{des}}$ are both known parameters that can be detected, we use the variable $\boldsymbol{\alpha}$ to refer to $\boldsymbol{E}_{M+1} \odot \left(\mathbf{I}-\mathbf{I}^{\mathrm{des}}\right)$ in subsequent calculations.

The gradient of the output light field with respect to the s_1^{th} neuron in the Mth layer, denoted as $\frac{\partial \boldsymbol{E}_{M+1}}{\partial \phi_{s_1}^{m}}$, can be obtained by taking the partial derivative:

$$\frac{\partial \boldsymbol{E}_{M+1}}{\partial \phi_{s_1}^{M}} = \mathbf{f'}_{\mathrm{M}} \odot \left(\mathbf{F}^{\dagger}\mathbf{P}\mathbf{F}\frac{d\Phi_M}{d\phi_{s_1}^{M}}\mathbf{E}_{\mathrm{M}}\right) \qquad (S-7)$$

where $\mathbf{f'}_{\mathrm{M}}$ is the derivative of the nonlinear activation function at the Mth hidden layer. Since the period of hidden layers is a fixed value, the diffraction matrix between any two hidden layers is the same and can be denoted as $\mathbf{P}$. For the convenience of calculation in simulation, a diagonal matrix $\boldsymbol{\Delta}_{\mathrm{s}_1}$ is introduced, and its diagonal elements are expressed as Eq. (S-8):

$$\boldsymbol{\Delta}_{\mathrm{s}_1}^{\xi,\xi} = \begin{cases} \mathrm{i}, & \xi = \mathrm{s}_1 \\ 0, & \text{otherwise} \end{cases} \qquad (S-8)$$

In this way, the gradient of the s_1th neuron in the Mth hidden layer can be expressed as Eq. (S-9):

$$\frac{\partial \boldsymbol{E}_{M+1}}{\partial \phi_{s_1}^{M}} = \mathbf{f'}_{\mathrm{M}} \odot \left(\mathbf{F}^{\dagger}\mathbf{P}\mathbf{F}\boldsymbol{\Phi}_{M}\boldsymbol{\Delta}_{\mathrm{s}_1}\mathbf{E}_{\mathrm{M}}\right) \qquad (S-9)$$

To improve the efficiency of numerical calculation, the light field vector is written as a diagonal matrix, and the diagonal elements are the elements in the corresponding position of the vector. At the same time, the derivative vectors of the nonlinear activation function are copied and combined to form a matrix. Then the gradient at the Mth hidden layer can be calculated according to Eq. (S-10):

$$\frac{\partial \boldsymbol{E}_{M+1}}{\partial \boldsymbol{\phi}_{M}} = \mathrm{i}\mathbf{f'}_{\mathrm{M}} \odot (\mathbf{F}^{\dagger}\mathbf{P}\mathbf{F}\boldsymbol{\Phi}_{M}\mathbf{E}_{\mathrm{M}}) \qquad (S-10)$$

Similarly, the gradient of the output light field with respect to the previous mth hidden layer can be expressed as Eq. (S-11):

$$\frac{\partial \boldsymbol{E}_{M+1}}{\partial \boldsymbol{\phi}_{m}} = \mathrm{i}\mathbf{f'}_{\mathrm{M}} \odot \{\mathbf{F}^{\dagger}\mathbf{P}\mathbf{F}\boldsymbol{\Phi}_{M} \ldots [\mathbf{f'}_{\mathrm{m}} \odot (\mathbf{F}^{\dagger}\mathbf{P}\mathbf{F}\boldsymbol{\Phi}_{m}\mathbf{E}_{\mathrm{m}})]\} \qquad (S-11)$$

Finally, the derivative of loss function L with respect to the mth hidden layer can be expressed as Eq. (S-12):

$$\frac{\partial \boldsymbol{L}}{\partial \boldsymbol{\phi}_{m}} = 4\Re\left\{\boldsymbol{\alpha}^{\dagger}\frac{\partial \boldsymbol{E}_{M+1}}{\partial \boldsymbol{\phi}_{m}}\right\}^{T} \qquad (S-12)$$

When training this theoretical model in a digital computer, the gradient information of the backpropagation can be obtained. However, the conditions of the actual experiments are not ideal, and the training of photonic chip does not strictly follow this theoretical model. In the training process of the actual experiment, the explicit gradient cannot be directly obtained, and we treat TDONN as a 'black box' with numerous trainable weight parameters. Since there is no explicit gradient, we update the weight parameters of the diffractive network by detecting the optical response of the output layer in real time. Specifically, in the TDONN prototype, the output of the photonic chip is detected by the PD array in real time, and the real-time cost function value is calculated as the evaluation index in the digital back-end. Then, the voltage applied to the on-chip diffractive unit is configured by the FPGA-based control framework to improve the cost function value, and finally the target function is realized.

Supplementary Note 4. Design of the period of hidden layers

In the TDONN architecture, the period of hidden layers, which is the spacing between adjacent hidden layers, is an important parameter that affects the diffraction of the light field in the slab waveguide and the computing accuracy. Before fabricating the TDONN chip, it is necessary to determine the

appropriate hidden layer period to ensure high accuracy. We conduct simulations using FPM and EBPM (mentioned in Supplementary Note 2 and Supplementary Note 3) to evaluate the impact of hidden layer period on the classification accuracy on multimodal datasets. In the simulation, the TDONN is used in four-class classification experiments. The dataset is divided into training set and test set (train:test =4:1), and the period is the only independent variable, which is set to 100, 120, 140, ..., 380, 400 μm, and other parameters remain the same. The classification accuracy corresponding to different period is obtained, and the results are shown in Supplementary Figure 3.

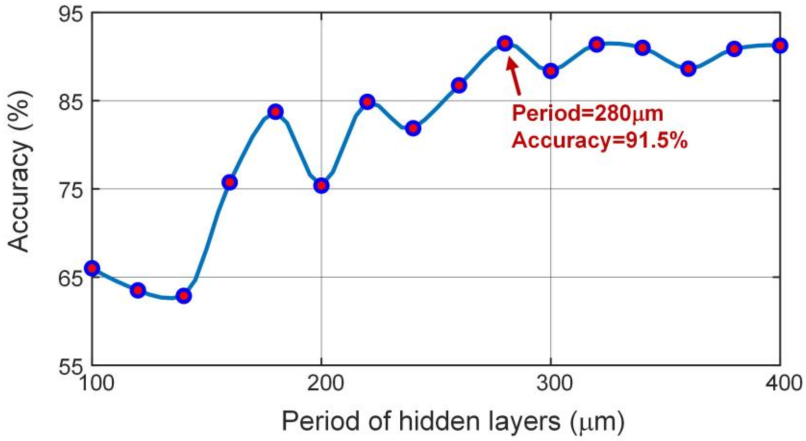

Supplementary Figure 3. Effect of the period on the classification accuracy of TDONN.

It can be observed that when the period is small, the accuracy of the test set increases as the period increases. After the period reaches 260 microns, the accuracy is more than 85%, and when the period =280 μm, the accuracy is the highest, reaching 91.5%. Tradeoff between integration density and accuracy, we choose period =280 μm as the design parameter of TDONN chip to achieve high accuracy at high integration density.

Supplementary Note 5. Simulation of the TDONN

The hidden layers of this model contain numerous tunable diffractive units. During the propagation of light in this model, each diffractive unit introduces a phase shift, and the phase shift will contribute to the modulation of optical signals. This is the basic principle and key concept of this model. In our proof-of-concept chip, phase shift is introduced by applying voltages on resistive heaters to change the temperature of the waveguide and cladding materials, thereby changing the effective refractive index. To visually show the effect of the voltage applied to the heater on the temperature, we simulate the thermal field of a single heater and multiple heaters when different voltages are applied.

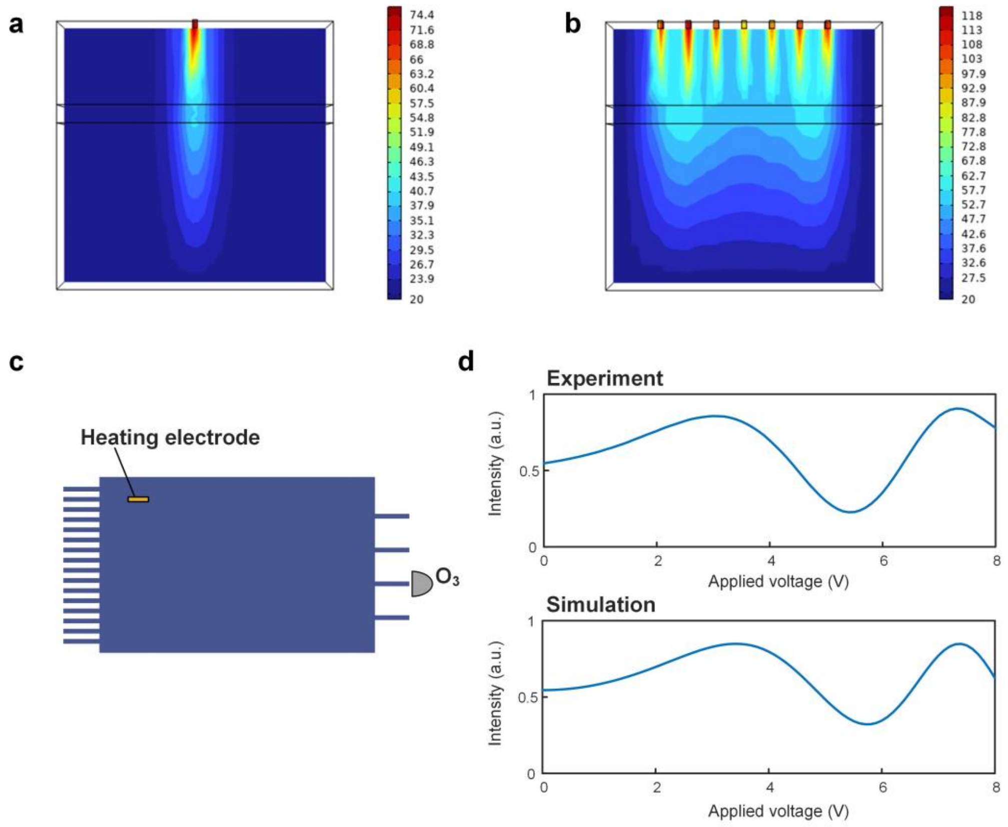

Supplementary Figure 4. Simulation of the TDONN. a Thermal field simulation of a single heater. **b** Thermal field simulation of multiple heaters. **c** Monitor the power of output port O_3 when tuning one heater. **d** Experiment and simulation results of output port O_3.

The thermal field simulation of a single heater is shown in Supplementary Figure 4a, and the thermal field simulation of multiple heaters is shown in Supplementary Figure 4b. To quantify the effect of applied voltages on the output of the TDONN chip, we perform further simulation and experiment. As shown in Supplementary Figure 4c, the monitor set at output port O_3 records the optical power when different voltages are applied to the heater. We test the effect of the on-chip diffractive unit on the output of TDONN by simulation and experiment respectively, and the results are shown in Supplementary Figure 4d. Simulation and experimental results show that the on-chip diffractive unit can effectively control the light field in TDONN, and verify agreement between TDONN's theoretical calculations and its physical field simulation.

Supplementary Note 6. Design details of the photonic-electronic prototype

With the continuous expansion of the scale of photonic integrated circuits, the number of on-chip modulation units is gradually increasing, which requires the multi-channel power supply and the precise control of each channel. To this end, we designed and developed a multi-channel power supply,

which is shown in the red dashed box in Supplementary Figure 5. The multi-channel power supply includes FPGA, ADC and DAC connected through the internal circuit to realize the required control functions. The DAC can be programmed to generate the required voltage (voltage range is 0~10V) for up to 96 channels. The ADC samples the output voltage of each channel at a 16-bit resolution to meet the precision demand. The FPGA, ADC, DAC circuits are designed as pluggable boards, thus the number of channels can be increased (or decreased) by plugging (or unplugging) pluggable boards according to specific experimental requirements.

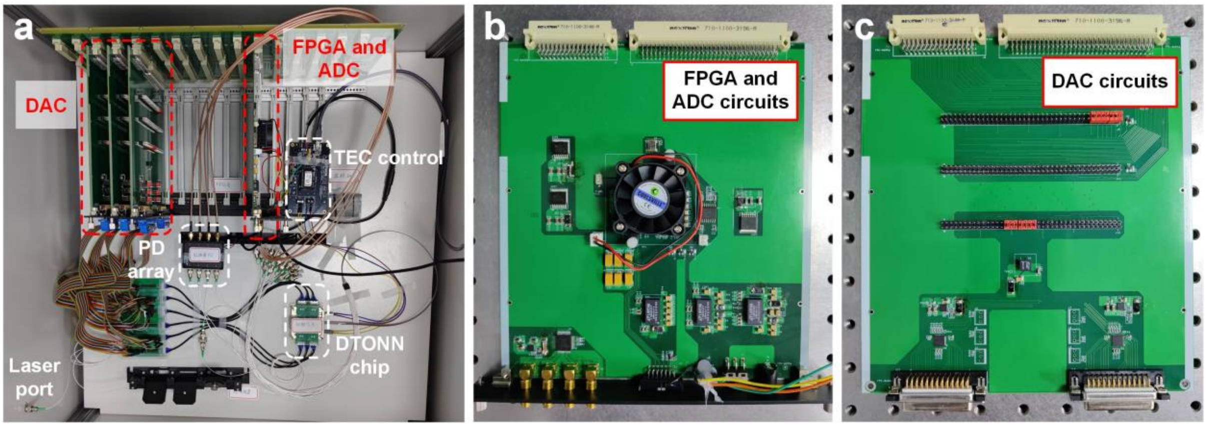

Supplementary Figure 5. Design of multi-channel power supply with FPGA, DAC, and ADC modules. a The multi-channel power supply (in the red dashed box). **b** The pluggable board designed with FPGA and ADC circuits. **c** The pluggable board designed with DAC circuits.

We further develop the photonic-electronic prototype and GUI software (see **Supplementary Movie 1-3**) based on this multi-channel power supply. In addition to the multi-channel power supply, the internal configuration also includes the adapter board, four-channel PD, temperature control module, polarization controller (PC) and the TDONN chip. The prototype has two primary interfaces: the data interface and the light source interface. The data interface facilitates information transmission and acquisition between the FPGA and the temperature control module. On the other hand, the light source interface establishes a connection for the local input to the external laser output. The work flow of the system unfolds in the following manner: the external light source enters the system through the light source interface, first passes through the PC and then coupled to the TDONN chip. The output data is received by the PD array and then passes to the FPGA through the ADC, and the FPGA receive the output data. After that, the FPGA provides the adjusted voltage information to the DAC module, and the procedure cycle is repeated to complete the training.

Supplementary Note 7. More tactile tasks for the TDONN chip

To better demonstrate the TDONN chip's capability, we further use the TDONN chip to classify gestures 5-8. Since the meaning of gestures 5-8 does not solely depend on the number of fingers, this classification task poses a greater challenge. Experimental results show that the trained TDONN chip can successfully recognize gestures 5-8, and the classification results are presented in Supplementary Figure 6.

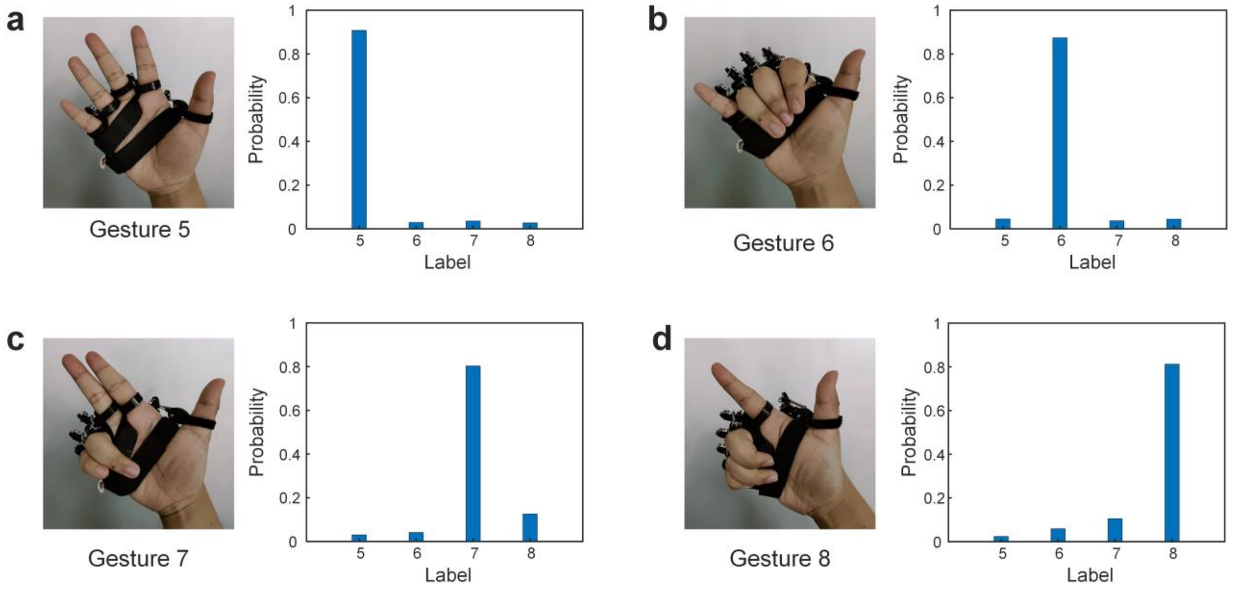

Supplementary Figure 6. Classification of gestures 5-8. a gesture 5, **b** gesture 6, **c** gesture 7, **d** gesture 8.

Supplementary Note 8. Estimation of the latency of the TDONN chip

The TDONN chip only needs one forward propagation of light to realize multimodal classification tasks, so its latency to complete a single task is very low. According to the estimation methods in related works[2-5], the latency of the TDONN chip is defined as the total time between the start of signal loading and the detection of the output, that is, the process does not include the input signal loading time and the output detection time. For the TDONN chip, the latency can be calculated by Eq. (S-13):

$$T_{delay} = D_1 \cdot \left(\frac{c_0}{n_{eff1}}\right)^{-1} + D_2 \cdot \left(\frac{c_0}{n_{eff2}}\right)^{-1} \qquad (S-13)$$

where D_1 is the distance from the narrow waveguide to the slab waveguide, D_2 is the length of slab waveguide, c_0 is the vacuum light speed, n_{eff1} is the effective refractive index (ERI) of the narrow waveguide, n_{eff2} is the ERI of the slab waveguide. Supplementary Figure 7 shows the micrograph of TDONN chip, and relevant parameters D_1 and D_2 in Eq. (S-13) are marked in the micrograph. Detailed

parameters include D_1=2.13 mm, D_2=1.35 mm, $n_{eff1} = 2.445$, $n_{eff2} = 2.848$, and the latency is about 30.2 ps.

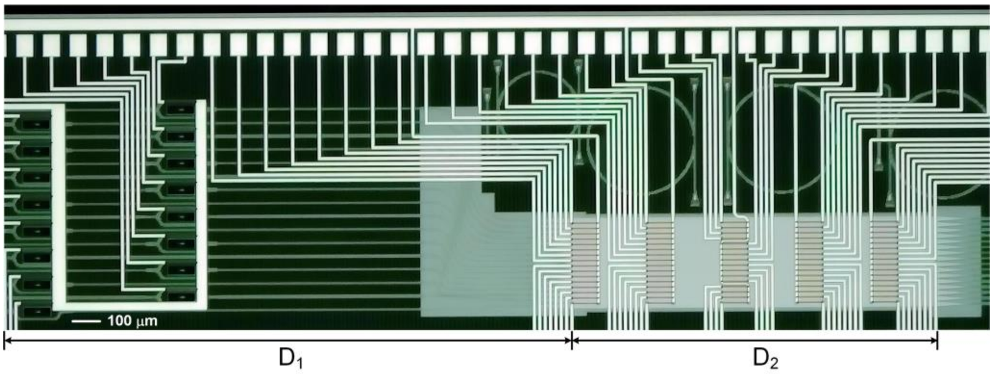

Supplementary Figure 7. The micrograph of TDONN chip. The relevant parameters D_1 and D_2 are marked in the micrograph.

Supplementary Table 1 shows the estimated latency of the prototype and provides the training and inference time required to complete a full task. The latency of the prototype system mainly consists of four parts: light propagation, response time of TiN heater, DAC, and ADC. The latency of light propagation is only 30.2 ps per iteration, and the operating frequency of the prototype is limited by the response time of TiN heater. To match the response time of thermo-optic modulation, the system operating frequency of the prototype is set to 10 kHz in multimodal task demonstration. The training process for completing a full task involves approximately 1000 iterations, and after training is completed, inference can be achieved with just one forward propagation. More importantly, the state of the heaters in the diffractive region of the TDONN chip need not be changed after the training is completed, so high-speed electro-optic modulators and PDs can be used for multimodal inference tasks to significantly reduce the latency.

Supplementary Table 1. Estimated latency of the TDONN prototype system.

**Parameters of the prototype system**	
Latency of light propagation	30.2 ps per iteration
Response time of TiN heater	10 μs per iteration
Latency of the DAC	0.1 ms per iteration
Latency of the ADC	0.1 ms per iteration
**Latency of training**	
Number of iterations required	1000
Total time	0.21 s
**Latency of inference**	
Number of iterations required	1
Total time	0.21 ms

To evaluate the relative efficiency of optical training, we train the same multimodal classification model using a digital computer (Intel(R) Core(TM) i9-12900K CPU, 32 GB RAM), and the time required for training is recorded. In the digital computer, it takes 1226.47 s to complete the training of the model. Under the operating frequency of 10 kHz, TDONN only needs 0.21 s to complete the training of the model, achieving more than 5800× acceleration.

Supplementary Note 9. Temperature stability of the TDONN

To verify the temperature stability of TDONN, an experiment is performed with and without the proposed drop-out method respectively, and the results are shown in Supplementary Figure 8. The blue line shows the results without the drop-out method, and the orange line shows the results with the drop-out method. It can be observed that the drop-out method helps to accelerate the training and achieve the target faster.

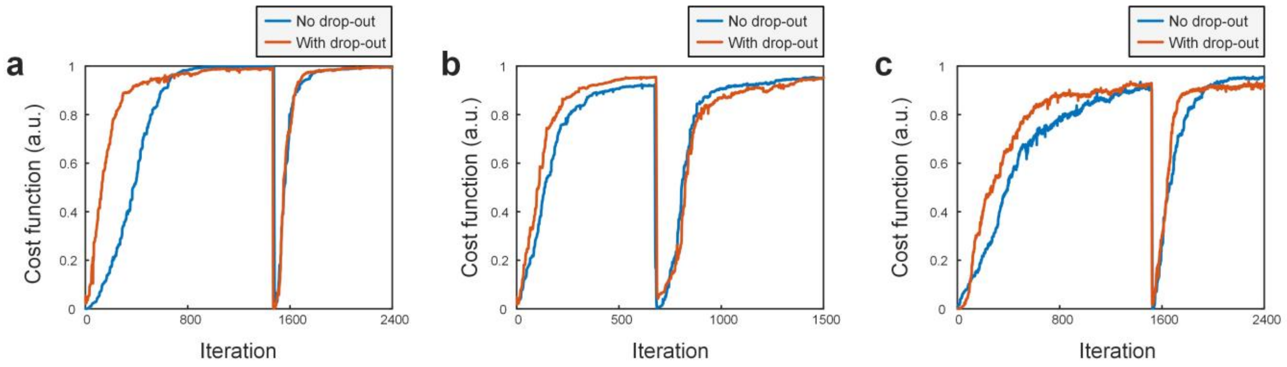

Supplementary Figure 8. Temperature stability test of the TDONN chip in multimodal tasks. a image classification, **b** audio classification, **c** tactile classification.

The temperature stability test is divided into three stages. The first stage is to optimize the TDONN chip at an initial temperature of 20 °C. With the increase of iterations, the cost function (CF) gradually converges to the maximum value until the function of the device meets the design goals. The second stage is used to demonstrate the degradation of device performance caused by external temperature changes. We use the temperature control module to gradually increase the external temperature of the TDONN chip from 20 to 30 °C. During the process of changing the temperature, the performance of the device begins to deteriorate significantly, and the CF value rapidly decays to near 0. The third stage is used to demonstrate the ability and robustness of the TDONN chip against environmental disturbances. The output of the chip is significantly abnormal due to external temperature changes, and the optimization program is activated and restarted. After several iterations at an external temperature of 30 °C, the CF value converges to the maximum value once again, indicating that only a short

optimization is required for the device function to meet the design goal even after a drastic change in external temperature.

Supplementary Note 10. Long-term stability of the TDONN

To verify the long-term stability of TDONN when performing classification tasks, an experiment is performed, and the results are shown in Supplementary Figure 9. The experiment consists of two stages: training and stability test. During the training stage, we train the TDONN chip to an optimal state using intelligent algorithms. In the stability test stage, we maintain the voltage applied to the on-chip trainable units and keep the trained TDONN prototype to operate for more than 6 hours. Throughout the experiment, we record the changes in the cost function over time. Since the time of stability test is much longer than the training time, to simultaneously show both the training and stability testing in the figure, we record the CF values in real-time during the training stage and once per second during the stability testing stage. Thanks to the TEC temperature control system and FPGA-based control framework, the TDONN prototype can maintain its original optimal state and successfully perform the target task even after 6 hours of completion of training. The experimental results indicate that the TDONN prototype exhibits excellent long-term stability.

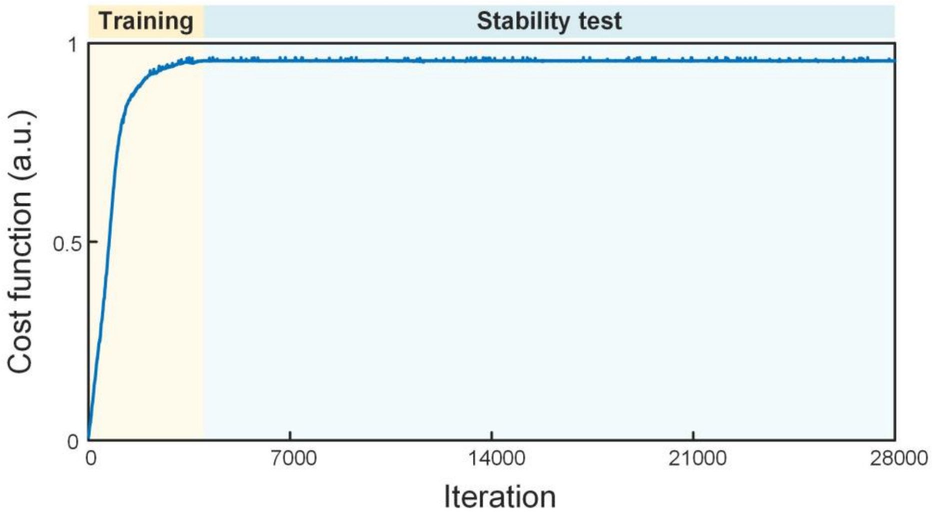

Supplementary Figure 9. Long-term stability test of the TDONN chip.

Supplementary Note 11. Estimation of throughput, computing density, and energy consumption

In the field of computing hardware performance evaluation, three critical metrics are throughput, computing density, and energy consumption. In the high-performance computing (HPC) domain, throughput is quantified as the number of operations per second (OPS) executed by a processor, serving

as a fundamental indicator of its performance. The throughput (T) for photonic computing hardware can be determined using Eq. (S-14)[6]:

$$T = 2m \times N^2 \times r\ OPS \qquad (S-14)$$

where T represents throughput in OPS (excluding off-chip signal loading time), m denotes the number of implemented layers in the photonic computing hardware, N^2 is the size of the on-chip weight bank, and r is the detection rate of the PDs. Given that the TDONN architecture inherently executes multiplication and accumulation (MAC) operations, where each MAC operation consists of a multiplication and an accumulation operation, one MAC operation corresponds to two operations.

Supplementary Table 2. Potential throughput estimation of the TDONN chip.

Layers	Scale	Potential throughput
1st layer	16×16	2×16×16×100 G=51.2 TOPS
2nd layer	16×16	2×16×16×100 G=51.2 TOPS
3rd layer	16×16	2×16×16×100 G=51.2 TOPS
4th layer	16×16	2×16×16×100 G=51.2 TOPS
5th layer with 4 outputs	16×4	2×16×4×100 G=12.8 TOPS
**Total layers**		**Total potential throughput**
Total 5 layers with 4 outputs		217.6 TOPS

Based on Eq. (S-14), we calculate the potential throughput for five hidden layers individually. The scale of the fifth hidden layer is 16×4, and the scale of other hidden layers is 16×16. At a typical frequency of 100 GHz, the potential throughput of each layer can be calculated. Summing the potential throughput of all five hidden layers, the total potential throughput of the TDONN chip can be obtained, which is 217.6 TOPS. Based on the calculated throughput, the computing density (C) of the TDONN architecture can be further determined through Eq. (S-15):

$$C = T/F \qquad (S-15)$$

where C represents the computing density, and F is the footprint of the photonic computing core. For our TDONN, the computing density is calculated to be 447.7 TOPS/mm². The power consumption of the TDONN encompasses both integrated chips and external benchtop instruments. Integrated chips include components such as data modulation, on-chip thermal phase shifters, PDs, thermoelectric coolers (TEC), and digital backends. External benchtop instruments comprise a laser source, arbitrary waveform generator and oscilloscope. Notably, previous works often exclude the power consumption of external instruments in their budgets. To facilitate a fair comparison, we calculate the expected power budget for integrated chips following methodologies in recent works[7-9]. The estimated total power consumption of the TDONN system is approximately 29.88 W, and the system-level energy

efficiency of the TDONN can be calculated as 7.28 TOPS/W. Notably, a substantial portion of the power consumption stems from the digital backend. When the power consumption of the digital backend module is not considered, the energy efficiency is 22.02 TOPS/W. The energy efficiency of the TDONN can be significantly improved by scaling up the on-chip diffractive network.

Supplementary Table 3. Estimated power consumption of the TDONN.

Module	Components	Power (W)
Laser source	DFB laser	0.5
Data modulation	Heaters (Input=16)	0.03×16=0.48
On-chip diffractive network	Heaters (N=80)	0.03×80=2.4
Photodetectors	PD driver (Output=4)	0.5×4=2
TEC	TEC for laser source<br>TEC for TDONN chip	1.5<br>3
Digital backend	FPGA control circuits	20
External benchtop instruments	Arbitrary waveform generator<br>Oscilloscope	50<br>120
Total power consumption<br>(include benchtop instruments)		199.88
Total power consumption<br>(exclude benchtop instruments)		29.88

Supplementary References

1. Khare K., Butola M., Rajora S. *Fourier optics and computational imaging*. Springer (2015).
2. Zarei S., Marzban M.-R., Khavasi A. Integrated photonic neural network based on silicon metalines. *Opt. Express* **28**, 36668-36684 (2020).
3. Fu T., *et al.* On-chip photonic diffractive optical neural network based on a spatial domain electromagnetic propagation model. *Opt. Express* **29**, 31924-31940 (2021).
4. Fu T., *et al.* Photonic machine learning with on-chip diffractive optics. *Nat. Commun.* **14**, 70 (2023).
5. Huang Y., Fu T., Huang H., Yang S., Chen H. Sophisticated deep learning with on-chip optical diffractive tensor processing. *Photonics Res.* **11**, 1125-1138 (2023).
6. Shen Y., *et al.* Deep learning with coherent nanophotonic circuits. *Nat. Photonics* **11**, 441-446 (2017).
7. Feldmann J., *et al.* Parallel convolutional processing using an integrated photonic tensor core. *Nature* **589**, 52-58 (2021).
8. Xu X., *et al.* 11 TOPS photonic convolutional accelerator for optical neural networks. *Nature* **589**, 44-51 (2021).

9. Bai B., *et al*. Microcomb-based integrated photonic processing unit. *Nat. Commun.* **14**, 66 (2023).
