# Supplementary material for: Multimodal deep learning using on-chip diffractive optics with in situ training capability
Source: Nat Commun. 2024 Jul 23;15:6189. doi: 10.1038/s41467-024-50677-3 (PMC11266606; doi:10.1038/s41467-024-50677-3)
Supplement: Supplementary file 3 — Description of Additional Supplementary Files [file 41467_2024_50677_MOESM3_ESM.pdf]

Description of Additional Supplementary Files

File Name: Supplementary Movie 1
Description: Optical inference with visual data.

File Name: Supplementary Movie 2
Description: Optical inference with audio data.

File Name: Supplementary Movie 3
Description: Optical inference with tactile data.
